# GPs’ drug treatment for depression by patients’ educational level: registry-based study

**DOI:** 10.3399/BJGPO-2020-0122

**Published:** 2021-02-10

**Authors:** Anneli Borge Hansen, Valborg Baste, Oystein Hetlevik, Inger Haukenes, Tone Smith-Sivertsen, Sabine Ruths

**Affiliations:** 1 Research Unit for General Practice, NORCE Norwegian Research Centre, Bergen, Norway; 2 Department of Global Public Health and Primary Care, University of Bergen, Bergen, Norway; 3 Division of Psychiatry, Haukeland University Hospital, Bergen, Norway

**Keywords:** antidepressive agents, depression, educational status, general practice, health services research, large database research

## Abstract

**Background:**

Antidepressant drugs are often prescribed in general practice. Evidence is conflicting on how patient education influences antidepressant treatment.

**Aim:**

To investigate the association between educational attainment and drug treatment in adult patients with a new depression diagnosis, and to what extent sex and age influence the association.

**Design & setting:**

A nationwide registry-based cohort study was undertaken in Norway from 2014–2016.

**Method:**

The study comprised all residents of Norway born before 1996 and alive in 2015. Information was obtained on all new depression diagnoses in general practice in 2015 (primary care database) and data on all dispensed depression medication (Norwegian Prescription Database [NorPD]) 12 months after the date of diagnosis. Independent variables were education, sex, and age. Associations with drug treatment were estimated using a Cox proportional hazard model and performed separately for sex.

**Results:**

Out of 49 967 patients with new depression (61.6% women), 15 678 were dispensed drugs (30.4% women, 33.0% men). Highly educated women were less likely to receive medication (hazard ratio [HR] = 0.93; 95% confidence interval [CI] = 0.88 to 0.98) than women with low education. No such differences appeared among men. Women aged 20–29 years were more likely to be treated with drugs than those aged 30–59 years, and women aged ≥70 years were more likely to receive drugs (HR = 1.65; 95% CI = 1.54 to 1.77) than those aged 20–29 years. The pattern was similar but less pronounced for men.

**Conclusion:**

Educational differences in antidepressant therapy among women may reflect different treatment approaches that clinicians should be aware of to avoid unintended variation. Reasons for this variation and consequences for quality of treatment should be explored.

## How this fits in

Medication for the treatment of depression is often prescribed by GPs, but little is known about factors that influence GP depression care. This study showed that highly educated women with a new depression diagnosis received less medication than women with low education, while no such differences appeared among men. Further, the youngest and oldest patients were most likely to receive antidepressant drugs. These differences may reflect different depression care approaches that clinicians should be aware of to avoid inequity in treatment.

## Introduction

The use of antidepressant drugs has increased substantially during the 1990s and early 2000s.^1–3^ With respect to prevalence of depression, studies indicate that women,^4,5^ older people,^6–8^ and lower socioeconomic groups^9^ are at increased risk. Moreover, women are more likely to consult their GP for depression compared with men.^10^ Whether drug use is equally distributed across population groups, such as higher and lower educated, is poorly examined and findings so far are inconsistent.^11–13^ With respect to sex and age, some studies indicate that GPs prescribe more depression drug therapy to women and older people than to men and younger people.^14,15^ However, there is a lack of studies that use a new depression diagnosis to confirm this trend.

GPs play a key role in providing health services to patients with depression. According to guidelines, talking therapy by GP is the first choice of treatment for mild depression.^16,17^ With increased severity, talking therapy may be combined with medication.^16^ In Norway, about 80% of antidepressant prescriptions are issued by a GP.^14^ Large registry-based studies with complete data on diagnoses, depression medication, and population demography may increase the knowledge and awareness about variation in healthcare provision to patients with depression.

The aim of this study was to investigate the association between educational attainment and drug treatment in adult patients with a new diagnosis of depression, and to what extent sex and age influence the association.

## Method

### Design

A nationwide registry-based cohort study was conducted with data from the Norwegian GP-DEP Study, which investigates pathways of depression care in general practice.^18^ The cohort comprised all individuals with a new depression diagnosis in general practice in 2015. The cohort was examined regarding dispensing of medication for depression in the 12 months after the first date of depression diagnosis (index date) in 2015.

### Data sources

Information from national registries was linked at the individual level, using the unique personal identity number (encrypted) assigned to all residents of Norway. All data were stored and analysed at a safe server at the University of Bergen.

The Control and Reimbursement of Healthcare Claims (KUHR) database stores data on all fee-for-service claims from GPs. For each encounter, the claims contain a GP and patient identifier, date of contact, and one or more diagnoses according to the International Classification of Primary Care, Second Edition (ICPC-2).

The NorPD stores information on all prescription drugs dispensed to patients treated in ambulatory care (www.norpd.no). For each prescription, NorPD contains an encrypted prescriber and patient identifier, date of dispensing, generic drug information (Anatomical Therapeutic Chemical [ATC] code), and any reimbursement code. NorPD lacks information at individual level on medication dispensed to people staying in hospitals or nursing homes.

The Norwegian Patient Registry (NPR) comprises information on all patient contacts with secondary health care, with diagnoses according to the International Classification of Diseases, Tenth Revision (ICD-10).

The National Education Database stores information on the highest level of completed education. While the population registry contains information on sex, year of birth, death, and emigration.

### Study population

**Figure 1.**
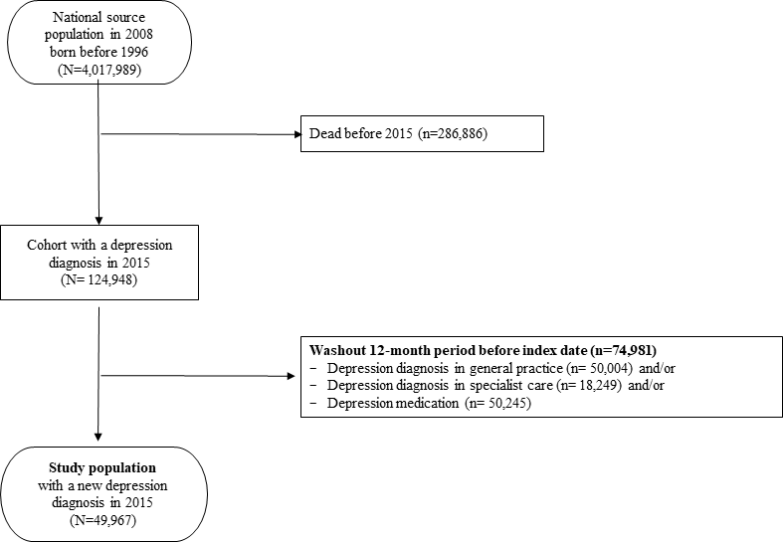
Flowchart illustrating the definition of the study population.

The source population comprised all inhabitants of Norway born before 1 January 1996 and alive 1 January 2015 (4 017 989 individuals). First, all individuals were identified with a depression diagnosis in general practice (GP consultation with the ICPC-2 code P76 Depression in KUHR) in 2015 (*n* = 124 948). Second, to establish a cohort of patients with a new diagnosis of depression, washout was conducted of 74 981 patients with a diagnosis of depression in general practice (P76 Depression in KUHR) and/or secondary care (ICD-10 codes F32, F33, F34, or F41.2 in NPR) and/or dispensed drug treatment for depression (NorPD) during 12 months before index date. The resulting study population comprised 49 967 individuals (Figure 1).

### Independent variables

The National Education Database is based on the International Standard Classification of Education.^19^ Eleven levels were recoded into three categories: low (primary school [grades 1–7] and lower-secondary school [grades ≤8–10]); medium (13 years, upper-secondary school); and high (>13 years, university and higher education). Patient age was recoded into decennial categories.

### Outcome

From NorPD all medications reimbursed for the treatment of depression were included: antidepressants (ATC code N06A), selected antiepileptic drugs (N03A), and selected antipsychotic drugs (N05A), dispensed during 12-month follow-up after index date (yes, no). Number of days from index date to first drug dispensing was categorised as 0–7, 8–31, 32–183, and 184–365 days. Since drug treatment was initiated by a GP in 86% of cases, the term 'GP drug treatment' was used.

### Statistical analysis

Descriptive statistics was used to examine the distribution of antidepressants, antiepileptics, and antipsychotics among the patients treated with drugs, given by numbers and percentages. Dispensing of medication and time interval from index date to first drug dispensing was provided by numbers and percentages, by educational level, sex, and age category. The associations between drug dispensing and education, sex, and age were examined by χ^2^ test. Further, Cox proportional hazard models were used to estimate the likelihood of being dispensed medication for the independent variables education, sex, and age. Interactions between education and sex, and between education and age, in the association with drug dispensing were tested in separate Cox proportional hazard models. Follow-up was defined in days from index date to first drug dispensing, and individuals were censored at the time of death, emigration, or end of follow-up, whichever occurred first. The results from the final model were presented stratified by sex (owing to interaction), both crude and adjusted, as HRs with 95% CIs. Reference groups were low education and age group 20–29 years.

The association between educational level and time to first drug dispensing was illustrated by Kaplan–Meier survival curves, and a pairwise log rank test was used to test the equality of the distribution of the survival curves between different education levels, and for sex separately. Missing data on education (1.1%) were excluded in the analyses. For all statistical analyses, *α* = 0.05 was used as significance level. IBM SPSS Statistics software was used (version 25).

## Results

The study population comprised 49 967 individuals with a new depression diagnosis in 2015, with a mean age of 44.4 years (standard deviation = 16.2 years); 30 775 (61.6%) women and 19 192 (38.4%) men. Among all patients, 30.1% had low education, 40.1% medium, and 28.1% high (Table 1). The study population comprised a relatively higher proportion of men and younger age groups compared with the washed-out population (see Supplementary Table S1).

**Table 1.** Drug treatment for patients with a new depression diagnosis in 2015, and time from date of diagnosis to first drug dispensing, by education, sex, and age

		**Patients treated with drugs for depression**				**Number of days from index date to first drug dispensing**			
		**No**		**Yes**		**0–** **7**	**8–** **31**	**32–** **183**	**184–** **365**
	**Total, *n***	***n***	**%**	***n***	**%**	**%**	**%**	**%**	**%**
**Educational level**									
Low	15 024	10 130	67.4	4894	32.6	17.8	4.4	7.1	3.3
Medium	20 015	13 624	68.1	6391	31.9	18.6	4.3	6.2	2.8
High	14 380	10 168	70.7	4212	29.3	16.1	3.9	6.5	2.7
Missing	548	—	—	—	—	—	—	—	—
**Sex**									
Women	30 775	21 421	69.6	9354	30.4	17.2	4.0	6.4	2.8
Men	19 192	12 868	67.0	6324	33.0	18.3	4.7	6.9	3.1
**Age group, years**									
20–29	10 975	7434	67.7	3541	32.3	17.0	4.6	7.3	3.4
30–39	10 157	7215	71.0	2942	29.0	14.9	4.2	6.9	3.0
40–49	10 951	7736	70.6	3215	29.4	15.2	4.5	6.8	2.9
50–59	8921	6326	70.9	2595	29.1	16.0	4.1	6.0	3.0
60–69	5136	3548	69.1	1588	30.9	19.9	3.4	5.6	2.0
≥70	3827	2030	53.0	1797	47.0	34.1	4.5	5.5	2.8
Total	49 967	34 289	68.6	15 678	31.4	17.6	4.3	6.6	2.9

Educational level: low (primary school [grades 1–7] and lower-secondary school [grades ≤8–10]); medium (13 years, upper-secondary school); and high (>13 years, university and higher education).

Among the study population, 15 678 (31.4%) were dispensed depression drugs during the 12-month follow-up, 9354 (30.4%) women and 6324 (33.0%) men (Table 1). Of those receiving medication, 85.2% were dispensed antidepressants only, 4.8% antipsychotics only, and 1.2% antiepileptics only, while 8.9% received drugs from two or three therapeutic groups (Table 2). Selective serotonin reuptake inhibitors (SSRIs) made up for 66.0% of the antidepressants (N06A) dispensed.

**Table 2.** Distribution of drug groups dispensed to patients with a new depression diagnosis in 2015 and treated with medication

**Drug group dispensed** ** **	***n***	**%**
Antidepressant drug only	13 356	85.2
Antipsychotic drug only	745	4.8
Antiepileptic drug only	183	1.2
Antidepressant + antipsychotic	1130	7.2
Antidepressant + antiepileptic	113	0.7
Antipsychotic + antiepileptic	56	0.4
Antidepressant + antipsychotic + antiepileptic	95	0.6
Total	15 678	100

Medications reimbursed for the treatment of depression in Norway (ATC code): antidepressants (N06A).

Non-selective monoamine reuptake inhibitors (N06AA): desipramine, imipramine, imipramine oxide, clomipramine, opipramol, trimipramine, lofepramine, debenzepin, amitriptyline, nortriptyline, doxepin, iprindole, melitracen, butriptyline, dosulepin, amoxapine, dimetacrine, amineptine, maprotiline, quinupramine. Selective serotonin reuptake inhibitors (N06AB): zimeldine, fluoxetine, citalopram, paroxetine, sertraline, alaproclate, fluvoxamine, etoperidone, escitalopram. Monoamine reuptake inhibitors (N06AG): moclobemide, toloxatone. Other antidepressants (N06AX): oxitriptan, tryptophan,mianserin, nomifensine, trazodone, nefazodone, minaprine, bifemelane, viloxazine, oxaflozane, mirtazapine, bupropion, medifoxamine, tianeptine, pivagabine, venlafaxine, milnacipran, reboxetine, gepirone, duloxetine, agomelatine, desvenlafaxine, vilazodone, hyperici herba, vortioxetine.

Antiepileptic drugs (N03A): valproic acid, carbamazepine, lamotrigine.

Antipsychotic drugs (N05A): ziprasidone, loxapine, olanzapine, quetiapine, asenapine, risperidone, aripiprazole, lithium.

Medication was more commonly provided to those with low educational level versus high, to men versus women, and to those aged 20–29 years or aged ≥70 years versus other age groups (Table 1). Altogether 8809 patients collected medication within 1 week of index date, corresponding to 56.2% (*n* = 8809/15 678) of patients treated with drugs, and 17.6% (*n* = 8809/49 967) of the total study population, respectively (data not shown).

Owing to a significant interaction between education and sex (*P* = 0.010), the Cox proportional hazard model was performed for men and women separately. Women with high and medium education were less likely to receive drugs (crude HR = 0.94; 95% CI = 0.90 to 0.99, and HR = 0.86; 95% CI = 0.81 to 0.90, respectively) compared with women with low education (reference), Table 3. The age-adjusted estimates were less pronounced but still significant for highly educated women. Among men there was no association between drug treatment and education. There was no interaction between sex and age. Women aged 20–29 years were more likely to be treated with medication compared with those aged 30–59 years, and women aged ≥70 years were even more likely to receive medication (HR = 1.65; 95% CI = 1.54 to 1.77) than those aged 20–29 years. The pattern was similar but less pronounced for men.

**Table 3.** Likelihood of receiving drug treatment among patients with a new depression diagnosis in 2015, by education and age; stratified by sex (*N* = 49 967)

		**Drug treatment**		**Unadjusted**		**Adjusted^a^**	
**Women**	**Total, *n***	***n***	**%**	**HR**	**95%** **CI**	**HR**	**95%** **CI**
**Educational level^b^**							
Low	8572	2775	32.4	1	—	1	—
Medium	11 829	3622	30.6	0.94	0.90 to 0.99	0.97	0.92 to 1.02
High	10 053	2844	28.3	0.86	0.81 to 0.90	0.93	0.88 to 0.98
**Age group, years**							
20–29	6802	2132	31.3	1	—	1	—
30–39	6190	1714	27.7	0.87	0.81 to 0.92	0.87	0.82 to 0.93
40–49	6577	1827	27.8	0.87	0.82 to 0.93	0.87	0.82 to 0.93
50–59	5338	1466	27.5	0.86	0.81 to 0.92	0.86	0.81 to 0.92
60–69	3161	969	30.7	0.99	0.92 to 1.07	1.00	0.92 to 1.07
≥70	2707	1246	46.0	1.66	1.66 to 1.55	1.65	1.54 to 1.77
**Men**							
**Educational level^b^**							
Low	6452	2119	32.8	1	—	1	—
Medium	8186	2769	33.8	1.04	0.98 to 1.02	1.04	0.98 to 1.10
High	4327	1368	31.6	0.96	0.90 to 1.03	0.97	0.90 to 1.04
**Age group, years**							
20–29	4173	1409	33.8	1	—	1	—
30–39	3967	1228	31.0	0.90	0.84 to 0.98	0.90	0.84 to 0.98
40–49	4374	1388	31.7	0.93	0.86 to 1.00	0.93	0.86 to 1.01
50–59	3583	1129	31.5	0.92	0.85 to 1.00	0.92	0.85 to 1.00
60–69	1975	619	31.3	0.94	0.85 to 1.03	0.94	0.85 to 1.03
≥70	1120	551	49.2	1.70	1.54 to 1.88	1.70	1.54 to 1.88

^a^Adjusted for age and education, respectively. ^b^Missing data on educational level; women (*n* = 321), men (*n* = 227). Educational level: low (primary school [grades 1–7] and lower-secondary school [grades ≤8–10]); medium (13 years, upper-secondary school); and high (>13 years, university and higher education).

**Figure 2.**
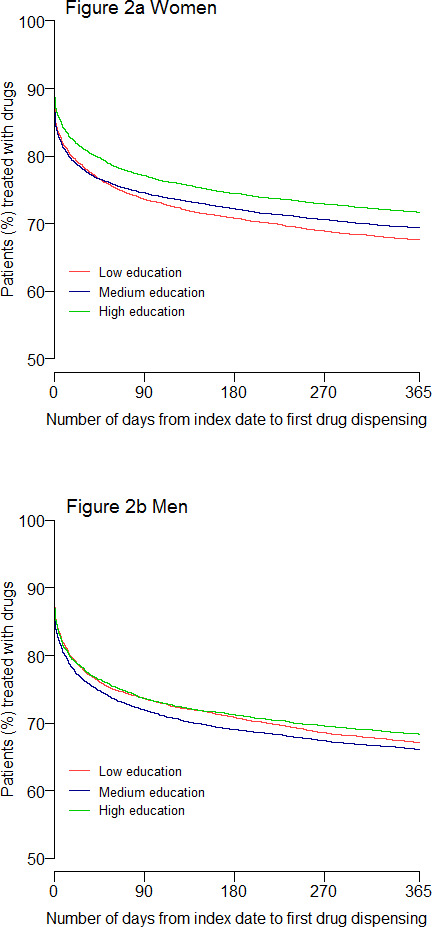
The association between education level and number of days from index date to first drug dispensing (Kaplan–Meier survival curves) for patients in Norway, aged ≥20 years with a new depression diagnosis in 2015 (*N* = 49 967).

Figure 2 illustrates the distribution of survival curves for time to first drug dispensing. Women (Figure 2a) with high education were dispensed depression drugs to a lesser extent and later after index date, compared with those with low or medium education (*P*≤0.001 for both groups). There was also a different distribution between medium and low educated women (*P* = 0.018). Highly educated men (Figure 2b) had a different distribution of drug dispensing than men with medium education (*P* = 0.013).

## Discussion

### Summary

In a nationwide cohort of patients with a new diagnosis of depression, a gendered pattern was found in the occurrence of depression and in the likelihood of receiving medication by educational level. While GP-diagnosed depression was considerably more prevalent among women, the proportion being treated with drugs was higher among men. Sex modified the relation between education and medication; hence, all analyses were performed separately for men and women. A novel finding was that highly educated women with new depression were significantly less likely to receive medication than lower educated women, even after adjusting for age. No educational differences were found for men. Finally, the youngest and the oldest patients were the age groups most likely to receive depression drugs.

### Strengths and limitations

The main strength of this study is the use of complete registry data from the publicly subsidised primary care services in Norway. Linkage of data from five national registries at the individual level provided a unique source of information, eliminating recall bias and selection bias.

Information on GP-diagnosed depression is another strength. A new depression diagnosis was defined as a GP consultation with the ICPC-2 code P76, after a 1-year washout period. However, the individual GPs set the diagnosis and the KUHR database has no formal control on diagnostic categories. Differing coding behaviour may, therefore, challenge the internal validity. However, potential misclassification by the GP would be non-differential and distributed randomly across population groups. Another limitation is lack of information on severity of depression, as ICPC-2 does not allow for such grading. Severity probably influences GPs’ decisions to initiate drug treatment. On the other hand, variation in severity is most likely also distributed evenly across patient educational level, sex, and age.

The NorPD contains complete data on all prescription drugs dispensed. Although the prevalence of prescribed medication for depression may have been slightly underestimated, the use of drug dispensing data is recognised as an acceptable proxy in epidemiological studies.^20^ Low out-of-pocket payment in Norway makes medication for depression easily available, and it is thus believed that primary non-compliance is low and evenly distributed across population groups. To strengthen the internal validity, drugs reimbursed for the treatment of depression have only been considered, eliminating for example, SSRIs for anxiety disorder and tricyclic antidepressants for adjuvant pain therapy.

### Comparison with existing literature

The prevalence of drug therapy for new depression found in the study (one of three patients) is considerably lower than antidepressant prescription rates of 45%–75% reported in studies from European countries.^21–23^ This discrepancy may be owing to use of a new diagnosis of depression, a strict definition of depression medication, and the use of drug dispensing data in the study. Half of the patients who started on drug treatment collected medication within 1 week after index date. This finding is in line with studies in the Netherlands and Sweden examining the time interval from depression diagnosis to initiation of drug treatment.^22,23^

Turning to sex, a Swedish registry study also including only newly diagnosed patients with depression found a slightly greater proportion of men than women receiving antidepressant drugs,^23^ in line with the present study's findings. The lower proportion of women using depression drugs may be related to gendered preferences; women preferring talking therapy, men drug treatment.^24–26^ On the other hand, GPs may initiate talking therapy to women owing to a preconception that they are more inclined to conversation.

A study in the UK among people aged ≥55 years showed that treatment rates with antidepressants were high for those recorded with a new depression diagnosis but varied little by age.^8^ The present study indicates that the youngest and oldest patients were most commonly prescribed medication. Higher prescription rates for older people may be an expression of unwarranted variation, since studies suggest that older people judge talking therapy more favourably than medication.^27,28^ GPs should be aware of older patients’ increased risk of polypharmacy and adverse side effects.^29^ This practice may nevertheless be owing to brief GP encounters with focus on somatic conditions,^27^ or to limited access to secondary mental health care.^30^

Depression is more prevalent among people with low socioeconomic status (SES) compared with those better off.^9^ Previous studies investigating the relationship between education and antidepressant use have demonstrated divergent findings. In line with the present study's results, a registry-based study in Sweden showed that poorly educated people received more prescriptions of antidepressants than those with higher education; the educational gradient being somewhat stronger among women than among men.^12^ Accordingly, Packness and colleagues in Denmark found that higher educated groups with no or few self-reported symptoms of depression were less likely to use medication; however, no associations were found among people with more pronounced symptoms.^13^ In contrast, Kivimäki and colleagues found lesser antidepressant use among men with lower education compared with men with higher education in Finland, while such differences were not seen in women.^11^ Finally, two studies conducted in Denmark and one in Australia showed no association between education and antidepressant use.^31–33^ The conflicting results in these studies could be owing to different study populations and measures of educational status, or to cross-national differences in access to treatment among disadvantaged people. In contrast to the current study, none of these studies comprised information on new depression diagnoses made by a GP.

Socioeconomic variation in antidepressant use found in this study may reflect an unintended bias by the GP letting SES influence prescription of medication,^33^ differential patient preferences supported by the GP, or a combination. One may speculate whether GPs prescribe drugs rather than provide talking therapy to patients they perceive as less educated, or whether better educated patients are more assertive about having non-pharmacological therapy. A survey among patients in GP waiting rooms in Norway showed that lower educational level was associated with greater preference for medication.^34^ The association between educational level and drug treatment found in the present study among women only, may suggest that highly educated women are more sceptical towards medication in general. This may apply particularly to younger women since older women in the study population were less educated.

### Implications for research and practice

The results of the study support an association between drug treatment for new depression and patient sex, education, and age. Highly educated women received less medication than women with low education, and no such differences appeared among men. Educational differences among women may reflect different treatment approaches that clinicians should be aware of to avoid unintended variation. Qualitative studies or register studies with long-time observation of treatment outcomes are needed to further explore the observed variation across educational levels, its reasons, and implications for the quality of depression treatment.
